# Editorial: Behavioral processes in online identity-related issues

**DOI:** 10.3389/fpsyg.2024.1446352

**Published:** 2024-06-21

**Authors:** Xi Chen, Ivan Wen, Qixing Qu, Wenjing Chen

**Affiliations:** ^1^Soft Science and Systems Science Research Center, Yunnan University, Kunming, China; ^2^Department of Management Science, Yunnan University, Kunming, China; ^3^Shidler College of Business, University of Hawaii at Manoa, Honolulu, HI, United States; ^4^School of Information Technology & Management, University of International Business and Economics, Beijing, China; ^5^School of Economics and Management, Beijing University of Posts and Telecommunications, Beijing, China

**Keywords:** behavioral processes, online identity, information security, identity construction and dynamics, social media

The advent of Internet-based new media has introduced a level of anonymity, making it a pivotal space for individuals to construct their identities. As media technologies advance, cyberspace is transitioning from a temporary escape to a new normal. Identity-related issues have emerged as a significant focus in interdisciplinary Internet research. Online identity, a complex social-psychological phenomenon, has garnered widespread attention from researchers across various fields. As we progress into the latter stages of Internet development, bolstered by emerging information technologies, digital media plays a crucial role in shaping identity, influencing online behavior, and guiding individuals to present specific personas. It is necessary to delve into the topics in the context of the behavorial processes in online identity related fields.

At first, privacy protection and information security in online identity construction. In social media, identity construction takes place through activities such as information sharing, self-disclosure, profile setting, and social interaction. These activities, however, can introduce significant privacy and security risks. The rapid dissemination of information heightens uncertainty, as the abstract nature of online identities and the widespread sharing of personal information make it difficult to distinguish between reality and fiction. The asynchronous and transcendent nature of online social interactions blurs the lines between private and public domains. Moreover, the simultaneous demands for justice and occurrences of online violence, such as doxing and online aggression, further threaten privacy and security. “Mindful sharenting” has become a strategy to balance sharing and protecting information, with privacy risks varying based on social context (Walrave et al.). Understanding these behaviors helps elucidate the strategies people use to protect privacy and security. Privacy concerns can inhibit social media participation, manifesting as lurking, fatigue, and self-withdrawal. Developing measurement tools is essential for quantitative research on these psychological manifestations (Chen and Yu). Misinformation is a significant challenge in the new media era, necessitating research into what motivates people to fact-check information and how they assess credibility, with cultural identities playing a notable role (Gottlieb et al.). Therefore, discussions surrounding privacy and information security protection must tackle the inherent advantages and threats that arise from the behavioral processes involved in online identity formation.

Next, identity construction and dynamics in online space. The online realm offers possibility for individuals to explore and express different aspects of their identity. Online interactions, feedback, and experiences can shape and alter an individual's digital identity over time. Social media platforms provide new tools and environments for identity formation and enactment (Bergs et al.). Influencers attract followers by presenting information (Tang et al.), and virtual idol fans actively participate in value co-creation, enhancing interpersonal interactions in online spaces (Wang et al.). This dynamic makes the identification between influencers and their audiences more robust in online space.

Additionally, functional support for specific identity. Online spaces provide and create connection mechanisms that enhance social accessibility for groups. This enables marginalized communities to find support and build connections with like-minded individuals (Xu and Zhang). Consumers can better meet their needs with the help of online information shaped by the consuming interaction model (Zhao et al.), fostering the formation of their identities. Emerging information technologies enhance the functionality of social media, with streaming content providing an immersive and vivid interactive experience, allowing different groups to realize their self-perception on platforms like TikTok (Zhu et al.). In the end, the functional support provided by social media has become an important mechanism for building different types of identities for various social groups across diverse fields.

Addressing the opportunities and challenges presented by identity issues in the virtual world, this Research Topic invites contributions from diverse conceptual and theoretical perspectives. The nine articles featured in this Research Topic delve into the topic from different angles, employing diverse methods including qualitative research, quantitative research, and mixed methods studies. Each approach showcases its unique strengths and insights. The theories employed in these studies span across various disciplines within the humanities and social sciences, highlighting the interdisciplinary nature of this topic. In today's increasingly complex online environment, multi-faceted discussions on online identity issues are of paramount importance. The editing of this Research Topic has been supported by the China National Social Science Fund project “Online Social Anonymity and Privacy Security Protection” (21BSH050). As online media enter the age of AI, there is no doubt that the construction of online identities and related research fields require further expansion and exploration.

## Author contributions

XC: Conceptualization, Funding acquisition, Supervision, Writing – original draft, Writing – review & editing. IW: Formal analysis, Investigation, Validation, Writing – review & editing. QQ: Investigation, Supervision, Validation, Writing – review & editing. WC: Supervision, Writing – review & editing, Resources.

